# CRF-Like Diuretic Hormone Negatively Affects Both Feeding and Reproduction in the Desert Locust, *Schistocerca gregaria*

**DOI:** 10.1371/journal.pone.0031425

**Published:** 2012-02-20

**Authors:** Pieter Van Wielendaele, Senne Dillen, Elisabeth Marchal, Liesbeth Badisco, Jozef Vanden Broeck

**Affiliations:** Molecular Developmental Physiology and Signal Transduction, Division of Animal Physiology and Neurobiology, Zoological Institute, K.U. Leuven, Leuven, Belgium; Arizona State University, United States of America

## Abstract

Diuretic hormones (DH) related to the vertebrate Corticotropin Releasing Factor (CRF) have been identified in diverse insect species. In the migratory locust, *Locusta migratoria*, the CRF-like DH (CRF/DH) is localized in the same neurosecretory cells as the Ovary Maturating Parsin (OMP), a neurohormone that stimulates oocyte growth, vitellogenesis and hemolymph ecdysteroid levels in adult female locusts. In this study, we investigated whether CRF-like DH can influence feeding and reproduction in the desert locust, *Schistocerca gregaria*. We identified two highly similar *S. gregaria* CRF-like DH precursor cDNAs, each of which also encodes an OMP isoform. Alignment with other insect CRF-like DH precursors shows relatively high conservation of the CRF/DH sequence while the precursor region corresponding to OMP is not well conserved. Quantitative real-time RT-PCR revealed that the precursor transcripts mainly occur in the central nervous system and their highest expression level was observed in the brain. Injection of locust CRF/DH caused a significantly reduced food intake, while RNAi knockdown stimulated food intake. Therefore, our data indicate that CRF-like DH induces satiety. Furthermore, injection of CRF/DH in adult females retarded oocyte growth and caused lower ecdysteroid titers in hemolymph and ovaries, while RNAi knockdown resulted in opposite effects. The observed effects of CRF/DH may be part of a wider repertoire of neurohormonal activities, constituting an integrating control system that affects food intake and excretion, as well as anabolic processes like oocyte growth and ecdysteroidogenesis, following a meal. Our discussion about the functional relationship between CRF/DH and OMP led to the hypothesis that OMP may possibly act as a monitoring peptide that can elicit negative feedback effects.

## Introduction

Insect diuretic neuropeptides stimulate primary urine secretion in the Malpighian tubules, the excretory organs. In many insect species, a diuretic hormone (DH) related to the vertebrate Corticotropin Releasing-Factor (CRF) has been characterized [1]. This CRF-like DH (CRF/DH) was first identified in the moth, *Manduca sexta* [2], but has now also been isolated and characterized in diverse insect species, such as the beetle *Tenebrio molitor* [3], the termite *Zootermopsis nevadensis* [4], the kissing bug *Rhodnius prolixus* [5] and the locust *Locusta migratoria* [6], [7]. In addition to its role in stimulating diuresis, the CRF-like DH was also suggested to be involved in the mediation of satiety in *L. migratoria*, since injection of this locust neuropeptide (*Locmi*-CRF/DH) influenced the duration of the meal and the latency to feed [8], [9]. Because *Locmi*-CRF/DH is progressively released during a meal [10], [11], it was suggested that the normal release of endogenous CRF-like DH signals the end of feeding [12]. The first precursor cDNA of a CRF-like DH was characterized in the moth *M. sexta* [13]. Subsequently, similar precursor sequences were found in other insect species by direct cloning or by blasting genome databases [14]–[17]. In locusts, this precursor had not yet been cloned or characterized.

By performing immunohistochemistry on *L. migratoria* brains, Tamarelle and coworkers showed that *Locmi*-CRF/DH is localized in the same neuroendocrine cells of the *pars intercerebralis* as the *L. migratoria* Ovary Maturating Parsin (*Locmi*-OMP) [18]. *Locmi*-OMP is a neurohormone of 65 amino acids (6.9 kDa), which stimulates oocyte maturation and vitellogenesis when daily injected in young adult female *L. migratoria* [19], [20]. These OMP injections also seemed to accelerate the occurrence of circulating ecdysteroids in the hemolymph [20], [21]. In the desert locust, *Schistocerca gregaria*, which belongs to the same family as *L. migratoria* (Acrididae, in the order of Orthoptera), two long and two short OMP-like molecules were purified and sequenced [22]. When injected in adult female *S. gregaria*, these *Schgr*-OMPs displayed biological activities similar to those of *Locmi*-OMP (*i.e.* acceleration of oocyte growth and appearance of circulating ecdysteroids). The two long *S. gregaria* isoforms only differ from each other by a tripeptide insertion/deletion at position 21 (Pro–Ala–Ala), while the two short isoforms are probably produced by truncation of these long isoforms [22]. Based on the restricted occurrence of OMP immunoreactivity in a few insect species and the observation that OMP has no sequence similarity with other known peptides, it was suggested that OMPs probably only occur in the order of Orthoptera, and more specifically in the family of Acrididae [23].

In the recently reported *S. gregaria* EST (“Expressed Sequence Tags”) database, the 5′ regions of two OMP-encoding precursor transcripts, each encoding one of the two large *Schgr*-OMP isoforms, were found [24]. By completing both sequences with 3′ Rapid Amplification of cDNA Ends (3′RAcE), we identified two full precursor sequences and discovered that each of these not only encode an *Schgr*-OMP isoform, but also contain the sequence of the locust CRF-like diuretic hormone. By means of quantitative real-time RT-PCR (qRT-PCR), the tissue distribution of these precursor transcripts was examined. In addition, RNA interference (RNAi), as well as peptide injections, were employed to further investigate the *in vivo* role of these precursor transcripts and their products in the regulation of food intake and female reproductive physiology. We were especially interested in the possible effects on oocyte growth and ecdysteroid levels, since it previously was described that OMP injection affects these parameters.

## Materials and Methods

### 1. Rearing of the animals

Adult female and male desert locusts [*Schistocerca gregaria* (Forskål)], were reared under crowded conditions at constant temperature (32+/−1°C) and constant day/night cycle (14 h photoperiod). They were fed daily *ad libitum* with fresh cabbage leaves and oat flakes. All used animals were synchronized on the day of their adult moult. Depending on the objective of the experiments, females and/or males were used. During each experiment, the different experimental groups were kept together in different compartments of the same cage which also contained a number of individuals of the opposite sex (in order to ensure a normal reproductive process and to prevent phase shifts) [25]. In the transcript profiling study, oocyte size (females) and cuticle colouration (males) were checked at the moment of dissection in order to rule out “underdeveloped” or “overdeveloped” animals.

### 2. RNA isolation and cDNA-synthesis

Desert locust tissues were dissected under a binocular microscope and rinsed in *S. gregaria* saline (1 L: 8.766 g NaCl; 0.188 g CaCl_2_; 0.746 g KCl; 0.407 g MgCl_2_; 0.336 g NaHCO_3_; pH 7.2). Afterwards, the tissues were immediately pooled in “MagNA Lyser Green Beads” containing tubes (Roche, Mannheim, Germany) and snap-frozen in liquid nitrogen to prevent degradation of the samples. The frozen samples were then stored at −80°C until further processing. To purify total RNA from these samples, they were first homogenized by placing the tubes into the MagNA Lyser instrument (30 s, 6500 rpm; Roche, Mannheim, Germany). Subsequently, total RNA was extracted from these samples by means of the RNeasy Lipid Tissue Mini Kit according to the manufacturer's protocol (Qiagen, Valencia, CA, USA). In combination with this extraction procedure, a DNase treatment (RNase-Free DNase set, Qiagen) was performed to eliminate potential genomic DNA contamination. The quality and concentration of the purified RNA was checked using a spectrophotometer (Nanodrop ND-1000; Thermo Fisher Scientific Inc., Waltham MA, USA). From each total RNA sample, cDNA was synthesized by reverse transcription of 1 µg of RNA with the Superscript® III Reverse Transcriptase (Invitrogen Life Technologies, Carlsbad, CA, USA) utilizing random hexamer primers according to the company's protocol. Before use, the resulting cDNA was diluted tenfold with Milli-Q® water and used to measure transcript levels by means of quantitative real-time RT-PCR.

### 3. Cloning of the *S. gregaria* OMP-DH precursors

In the *S. gregaria* EST database [24], two partial OMP-precursor-encoding sequences, consisting of the 5′ part of these transcripts, were found. By sequencing the corresponding plasmids, both sequences were verified. To obtain the remaining part of both precursor-encoding sequences, a 3′RAcE procedure (Rapid Amplification of cDNA Ends) was performed, by means of the SMART™ RACE cDNA Amplification Kit (Clontech Laboratories, Mountain View, CA, USA) and according to the manufacturer's protocol. The starting material for this procedure was an RNA sample derived from brains of females (day 10 of adult stage). Because of the high GC-content of the known part of the precursors (and the low specificity of the PCR amplification) a nested PCR was performed. The following gene-specific primers were used: 5′-AGGACAATGTCGCCGGTGAGAGTG-3′ and 5′-CGGCTGCTCTGCCTACTACGAG-3′ (Sigma–Aldrich, St. Louis, MO, USA), the second one being the “nested” primer. Since the amplification efficiency and specificity were too low when using the Advantage Polymerase Mix (from the kit) or other Taq polymerases, the Pwo SuperYield DNA Polymerase (Roche) was used, together with the GC-RICH Resolution Solution (supplied with the Pwo Polymerase kit) to enhance the amplification. The reaction mixture contained 5 µl Pwo buffer, 1 µl dNTP solution (10 mM for each nucleotide), 0.5 µl Pwo Polymerase, 10 µl GC-Rich resolution solution, 3.5 µl cDNA, 1 µl Gene-specific primer, 5 µl of the Universal Primer Mix (provided in the used RACE kit) and 24 µl Milli-Q® water. For the nested PCR, a similar reaction mixture was used but with 28 µl Milli-Q® water and 1 µl of the nested Universal primer A (instead of the Universal Primer Mix). The following thermal cycling profile was used for the first PCR reaction: initial denaturation at 95°C for 2 min, followed by 5 cycles at 95°C (30 sec) and 72°C (3 min), then five cycles at 95°C (30 sec), 70°C (30 sec) and 72°C (3 min), followed by 30 cycles at 95°C (30 sec), 68°C (30 sec), and 72°C (3 min). The PCR program was ended with a final extension step at 72°C (3 min). The PCR reaction was diluted 200-fold in Tricine-EDTA Buffer supplied in the SMART™ RACE Kit and used as template for the second “nested” PCR reaction using the following thermal cycling profile: initial denaturation at 95°C (2 min), followed by 30 cycles at 95°C (30 sec), 68°C (30 sec) and 72°C (3 min). The products of the PCR reaction were analyzed with agarose gel electrophoresis and extracted from the gel with the GenElute Gel Extraction Kit (Sigma-Aldrich). Since PCR products produced by amplification with Pwo polymerase do not contain 3′-A overhangs (necessary for the subsequent cloning step), the extracted fragments were incubated at 72°C (15 min) with dATPs and Taq DNA Polymerase (Sigma-Aldrich). The following reaction mixture was used: 2 µl 10× Taq Polymerase Buffer, 1 µl dATP (2 mM), 1 µl MgSO4 (50 mM), 0.5 µl Taq Polymerase (Fermentas), 10.5 µl Milli-Q® water and 5 µl of the extracted PCR products. The DNA fragments were subsequently cloned into the pCR®4-TOPO® vector by means of the TOPO TA Cloning® Kit for Sequencing (Invitrogen). The sequences of the inserted DNA fragments were determined using the ABI PRISM BigDye Terminator Ready Reaction Cycle Sequencing Kit (Applied Biosystems).in combination with the ABI PRISM 3130 Genetic Analyzer (Applied Biosystems), according to the manufacturer's protocol. Both obtained full precursor cDNAs have been deposited in GenBank under accession numbers JN591547 (OMP-DH-L precursor transcript) and JN591548 (OMP-DH-S precursor transcript).

### 4. Quantitative real-time PCR

Quantitative real-time PCR (qRT-PCR) reactions were performed according to the Fast SYBR Green PCR Master Mix protocol (Applied Biosystems, Carlsbad, CA, USA), in a 20 µl reaction volume, containing 5 µl of the previously diluted cDNA samples. The primers for the *Schgr*-OMP-DH target transcripts were designed by means of the Primer Express software package (Applied Biosystems). Due to the very high sequence similarity of both OMP-DH precursor transcripts, no primer pairs specifically recognizing one of both transcripts could be designed. Therefore, we chose to use one primer pair recognizing both transcripts. The final concentration of the primers in the reaction mix was 300 nM. The reactions were run in triplicate on a StepOnePlus™ Real-Time PCR System (Applied Biosystems) applying the following thermal cycling profile: holding stage at 95°C (10 min), followed by 40 cycles of 95°C (3 s) and 60°C (30 s). Amplification data were analyzed by means of the StepOne Software v2.0 (Applied Biosystems). The measured levels of the target transcripts were normalized according to the geNorm method [26], [27]. Accurate normalization of qRT-PCR data is necessary to compensate for possible variations due to imperfections in reverse transcription. For each qRT-PCR-experiment, suitable reference genes were selected from a pool of candidate reference genes. This was done by means of the geNorm Excel applet [26] based on the measured expression levels of these genes in the different samples. The seven candidate reference genes were EF1α (elongation factor 1 alpha), Rp49 (ribosomal protein 49), GAPDH (glyceraldehyde 3-phosphate dehydrogenase), β-actin, α-tubulin, ubiquitin and CG13220 (the *S. gregaria* homolog of the reference gene CG13220 used in *D. melanogaster*) [27]. The primers for these genes are represented in Table 1. For the qRT-PCR experiments described in this paper, the geNorm applet selected RP49 and EF1α as suitable reference genes.

**Table 1:** Oligonucleotide primers used in quantitative real-time RT-PCR.

Transcript	Forward primer (5′→3′)	Reverse primer (5′→3′)
β-Actin	AATTACCATTGGTAACGAGCGATT	TGCTTCCATACCCAGGAATGA
Rp49	CGCTACAAGAAGCTTAAGAGGTCAT	CCTACGGCGCACTCTGTTG
EF1α	GATGCTCCAGGCCACAGAGA	TGCACAGTCGGCCTGTGAT
GAPDH	GTCTGATGACAACAGTGCAT	GTCCATCACGCCACAACTTTC
Ubiquitin	GACTTTGAGGTGTGGCGTAG	GGATCACAAACACAGAACGA
α-Tubulin	TGACAATGAGGCCATCTATG	CGCAAAGATGCTGTGATTGA
CG13220	TGTTCAGTTTTGGCTCTGTTCTGA	ACTGTTCTCCGGCAGAATGC
OMP-DH-P	GCCTGTACGGCCACCTGTT	TCTCACCGGCGACATTGTC

Abbreviations used: Rp49, ribosomal protein 49; EF1α, elongation factor 1 alpha; GAPDH, glyceraldehyde 3-phosphate dehydrogenase; CG13220, *S. gregaria* homolog of the reference gene CG13220 used in *D. melanogaster*; OMP-DH-P, OMP-DH precursors from *Schistocerca gregaria* (described in this study).

To confirm the specificity of the PCR reactions, dissociation curves were analyzed showing a single melting peak. Additionally, amplification products of PCR reactions were run on an agarose gel by electrophoresis. Visualization of the PCR products showed the presence of a single band of the expected size for each transcript. Sequencing of these PCR products further verified the specificity of the qRT-PCR amplification. “No template control” reactions showed there was no contamination with foreign DNA. To correct for differences between different qRT-PCR runs, PCR reactions were also performed with a calibrator sample. For each experimental condition, samples of three biologically independent pools were measured. In order to test the statistical significance of the observed differences, linear regression analysis was performed using the GraphPad Prism 5 program (GraphPad Software Inc.) for the RNAi knockdown experiments.

### 5. Design of the CRF/DH-injection experiments

Female adults were taken on the day of final moult and injected daily until day 15 of the adult stage using a 710RN 100 µl Syringe (Hamilton, Bonaduz, Switzerland). Injections were performed directly in the hemocoel between the first and second abdominal segments in the direction of the head. Control animals were injected with 4 µl Milli-Q® water, while the experimental animals were injected with 1 pmol CRF-like DH dissolved in 4 µl Milli-Q® water. At several time points during the experiments, hemolymph samples were taken and ecdysteroid levels were determined by means of enzyme immunoassay (EIA, see “Materials and Methods”: section 9). On day 15, oocyte size was measured (see “Materials and Methods”: section 9) and ovaries were dissected and prepared for determination of ecdysteroid levels by means of EIA.

### 6. RNA interference experiments

#### Production of dsRNA

dsRNA was produced with the MEGAscript® RNAi Kit (Ambion, Austin, TX, USA). Sense and anti-sense RNA strands were produced in separate reactions. A part of the shortest OMP-DH precursor cDNA was used as template. This target region is, except for the 9 bp insertion and 1 point mutation in the OMP-coding region, identical to the corresponding part of the other precursor. As a result of this, the produced dsRNA is expected to knock down both precursors at the same time. This cDNA fragment was inserted into the pCR™4-TOPO® vector, in two different orientations with respect to a T7 promotor. In this way, sense and anti-sense strands could be transcribed by the T7 RNA polymerase (provided in the kit). In order to enhance the efficiency of the dsRNA production, the constructs were linearized (Figure S1). After *in vitro* transcription, both reaction mixtures were combined for annealing and purification. For control conditions, Green Fluorescent Protein (GFP) dsRNA was produced in the same way.

A 524 bp part of the shortest OMP-DH precursor was amplified from cDNA (derived from pooled adult female brains) by means of a PCR reaction using the Pwo Superyield DNA Polymerase (Roche) and the GC-Rich resolution solution (provided with the Pwo Polymerase), according to the manufacturer's instructions. For the production of the GFP dsRNA, a 589 bp partial GFP sequence (not containing introns) was amplified from a pMT/BiP/V5-His/GFP vector in the same way. The primers used for these procedures are shown in Table 2. All primers contained a unique restriction site in order to specifically cleave the constructs at the correct side of the inserts (Figure S1). In both cases, the following thermal cycling profile was used: initial denaturation at 95°C (2 min), followed by 34 cycles at 95°C (30 s), 60°C (30 s) and 72°C (2 min 30 s). The PCR program ended with a final extension step at 72°C (2 min). The PCR reactions were analyzed by means of agarose gel electrophoresis, extracted from the gel, prepared for cloning (3′A overhangs) and cloned into the pCR™4-TOPO® vector with the TOPO TA Cloning Kit (Invitrogen), as described in point 3 of “Materials and Methods”. By sequencing the constructs from the different colonies, the sequence and orientation of the inserted fragment were checked. A “sense” and “anti-sense” construct were selected, grown up in bacterial cells and again purified with the EndoFree Plasmid Maxi Kit (Qiagen). Next, they were digested with FastDigest restriction enzymes (Fermentas UAB, Vilnius, Lithuania). The following enzymes were used: BclI for the OMP-DH sense construct, PvuII for the OMP-DH anti-sense construct, SnaBI for the GFP sense construct and PmlI for the GFP anti-sense construct. All digestion reactions were performed according to the manufacturer's instructions and checked by agarose gel electrophoresis.

**Table 2:** Oligonucleotide primers used in this study for the production of dsRNA, directed against the *Schgr* OMP-DH precursors (OMP-DH) or against a GFP sequence (GFP).

Target	Forward primer (5′→3′)	Reverse primer (5′→3′)
OMP-DH	**CAGCTG**CTCTGCCTACTACGAG	**TGATCA**GATTTCAAGAATGCTTACGA
GFP	**CACGTG**AAGGTGATGCTACATACGGAA	C**TACGTA**ATCCCAGCAGCAGTTACAAAC

The bold values in the primer sequences represent restriction sites (OMP-DH forward-primer: PvuII, OMP-DH reverse-primer: BclI, GFP forward-primer: PmlI, GFP reverse-primer: SnaBI).

The actual production of the dsRNA (transcription, annealing and nuclease digestion) was performed using the ‘MEGAscript® RNAi Kit’ (Ambion) according to the manufacturer's instructions. The sense and anti-sense constructs were transcribed in separate reactions, after which the sense and anti-sense strands were combined for annealing. For this, the mixture was heated to 75°C for 5 minutes and subsequently cooled to room temperature. To eliminate potential ssRNA and the still present construct DNA, a nuclease digestion with RNase and DNase I (both supplied with the kit) was performed. The dsRNA was purified out of the reaction mixture with a phenol/chloroform extraction followed by an ethanol precipitation. The dsRNA pellet was redissolved in the elution buffer provided in the MEGAscript® RNAi Kit. The quality and concentration of the produced dsRNA was determined by means of spectrophotometry (Nanodrop ND-1000). To check the integrity of the dsRNA, a small amount of the reaction product was analyzed by agarose gel electrophoresis.

#### RNAi-experiments

Adult females were synchronized on the day of their final moult (day 0). The animals were injected using a 710RN 100 µl Syringe (Hamilton, Bonaduz, Switzerland). Injections were performed directly into the hemocoel between the first and second abdominal segments in the direction of the head. All animals received 4 µg dsRNA, OMP-DH dsRNA for the experimental animals, GFP dsRNA for the control animals. Preliminary experiments were performed to determine the optimal dose of dsRNA to be injected (unpublished results). Subsequently, the knockdown efficiency was measured in brains, optical lobes and suboesophageal ganglia at five and seven days after injection in order to further verify the knockdown effect in females (Figure S2) and males (data not shown). At both time points, the OMP-DH transcripts were strongly downregulated in the three analyzed tissues (>90% knockdown). The qRT-PCR amplicon did not overlap with the transcript region complementary to the produced dsRNA. Because of this, we only measured transcript levels and not levels of dsRNA in order to make correct estimates of the knockdown efficiency.

### 7. Measuring oocyte size

For every animal, the length of 10 terminal oocytes (i.e. oocytes at the base of the ovarioles and hence the largest oocytes) was measured with a piece of millimeter-squared paper. The average of the different measured oocytes was calculated. In order to test the statistical significance of the observed differences, a One-way ANOVA-test was performed using the Statistica software (StatSoft, Tulsa, OK, USA), after checking the validity of the assumptions on which this test is based.

### 8. Measuring food intake

The effects of CRF/DH-injection, knockdown of the OMP-DH precursors and rescue of this knockdown by CRF/DH injection, were analyzed on food intake. For this, animals received a dsRNA injection on day 5 (OMP-DH dsRNA or GFP dsRNA) as well as an injection of the CRF/DH (dissolved in Milli-Q® water) or an injection with Milli-Q® water on day 10, after which food intake was measured. The injected CRF-like DH of *L. migratoria* is 100% identical to the CRF-like DH of *S. gregaria* (see “Results” section) and was a kind gift from Dr. Orchard (Toronto, Canada).

When measuring the effect of a treatment on food intake, it needs to be ensured that all animals are in the same “feeding state”. Prior to the dsRNA injection on day 5, animals were fed. After the injections, animals were returned to their cages but they did not receive food for 24 hours. After this period, they were fed until all animals had finished eating. This was checked by temporarily removing the “fed” animals from the cage. After this short feeding period, the food was taken away and the animals were starved for 3 days. After this starvation period, the animal's appetite was checked by measuring the amount of cabbage leaf eaten by the individual animals (each in a separate cage) during one meal. This was done by measuring the weight of the leaves before and after the meal, taking into account the evaporation of the leaves. One meal was defined as the consumption of food from the moment the animals started feeding (immediately after they received food) until they migrated away from the food (the animals did not restart feeding in this assay). The leaf parts that were used, were originating from the same cabbage leave and were similar in size, weight and quality (*e.g.* similar venation). Prior to this meal, animals were injected with Milli-Q® water or CRF-like DH (0.8 pmol in 4 µl Milli-Q® water) and placed individually in a small cage. They were allowed to habituate to their new cage for 15 min. Next, they received the leaves and were allowed to eat. After 30 minutes, the food was removed and weighed. This 30 minute period was shown to be sufficient for one meal, since all animals had moved away from the food by then and had not yet restarted feeding. In order to correct for the animal's body size, animals were weighed immediately prior to the experiment and total food intake was calculated relative to the animal's body weight. Each condition consisted of 15 males and 15 females. The different experimental groups were analyzed by means of a One-way ANOVA-test, using the Statistica software.

### 9. Ecdysteroid extraction and quantification

#### Ecdysteroid extraction of hemolymph samples

5 µl hemolymph samples from adult females were taken at different time points during the RNAi experiment. Each sample was collected in 100 µl ice-cold methanol and stored at −20°C until further processing. The samples were extracted three times with 100% methanol by cyclic centrifugation and collection of the supernatants. The different supernatants originating from the same sample were combined and dried in a vacuum centrifuge. When completely dry, the samples were dissolved in a sample buffer for Enzyme Immuno Assay (EIA) measurement (0.1 M phosphate buffer, pH 7.4) and stored at −20°C.

#### Ecdysteroid extraction out of ovary samples

Complete ovaries were dissected, carefully rinsed in Ringer solution, placed in ice-cold pure methanol and kept at −20°C until further processing. The tissues were processed as described by Tawfik and colleagues [28]. Ovaries were homogenized in methanol by means of a sonicator. The samples were then heated to 60°C for 10 min followed by centrifugation at 10,000 g (10 min). The supernatants were collected and the pellets were re-extracted twice. The different supernatants originating from the same sample were combined and dried by evaporation in a vacuum centrifuge. Apolar lipids were removed by dissolving the sample in hexane and 70% methanol followed by mixing, centrifuging and discarding the upper hexane phase. The remaining methanol phase was divided in two equal halves. Both halves were desiccated with the vacuum centrifuge. One of them was dissolved in the sample buffer for EIA measurement. The other half was subjected to enzymatic treatment. A large portion of the ecdysteroids present in locust eggs is conjugated. This conjugation hampers binding of the ecdysteroids to the antibody used in the EIA measurement. The enzymatic treatment converts conjugated ecdysteroids into “free” ecdysteroids which can be recognized by the antibody used in the EIA. The sample is dissolved in 2 ml sodium acetate buffer (50 mM, pH 5.1). This buffer volume contained 1 mg type H-1 β-glucuronidase/arylsulphatase l from *Helix pomatia* (Sigma, St. Louis, MO, USA) and 1 mg type I1 acid phosphatase from potatoes (Sigma). The samples were incubated at 37°C for 24 h. Afterwards, reactions were terminated by adding pure methanol and dried with the vacuum centrifuge. The desiccated samples were then dissolved in sample buffer and measured with the enzyme immunoassay.

#### Ecdysteroid quantification

Ecdysteroid levels were evaluated using enzyme immunoassay (EIA), according to the method of Porcheron *et al.* [29], modified by using a peroxidase conjugate of 20-hydroxy-ecdysone as tracer and a rabbit polyclonal antibody (L2) against ecdysteroids [30]. The antibody and tracer were a kind gift from Dr. De Reggi (Marseille, France) and Dr. Delbecque (Bordeaux, France). After incubation to allow the immunological reaction to take place and subsequent washing steps, a coloration reaction was started by the addition of UHP (urea-hydrogen peroxide adduct, Sigma) and TMB (tetramethylbenzidine, Sigma). Absorbance was measured every 5 minutes at 370 nm for one hour. In order to quantify the ecdysteroid levels, a serial dilution (ranging from 10^−8^ M till 10^−12^ M) of ecdysone (E) or 20-hydroxy-ecdysone (20E) was placed on each 96-well plate. Results were determined by comparison with dose-response curves obtained using these diluted standards and were calculated as E equivalents or 20E equivalents. For the ovary samples, ecdysone was used as standard since it was shown to be the main ecdysteroid in adult ovaries of *S. gregaria* [28]. Although ecdysone and 20-hydroxy-ecdysone are similarly abundant in female adult hemolymph samples [31], 20-hydroxyecdysone was chosen as standard because of its general physiological importance. In order to test the statistical significance of the observed differences, unpaired t-tests were performed using the Statistica program (Statsoft), after checking the validity of the assumptions on which this test is based.

## Results

### 1. cDNA sequences of the OMP-DH precursors

**Figure 1:**
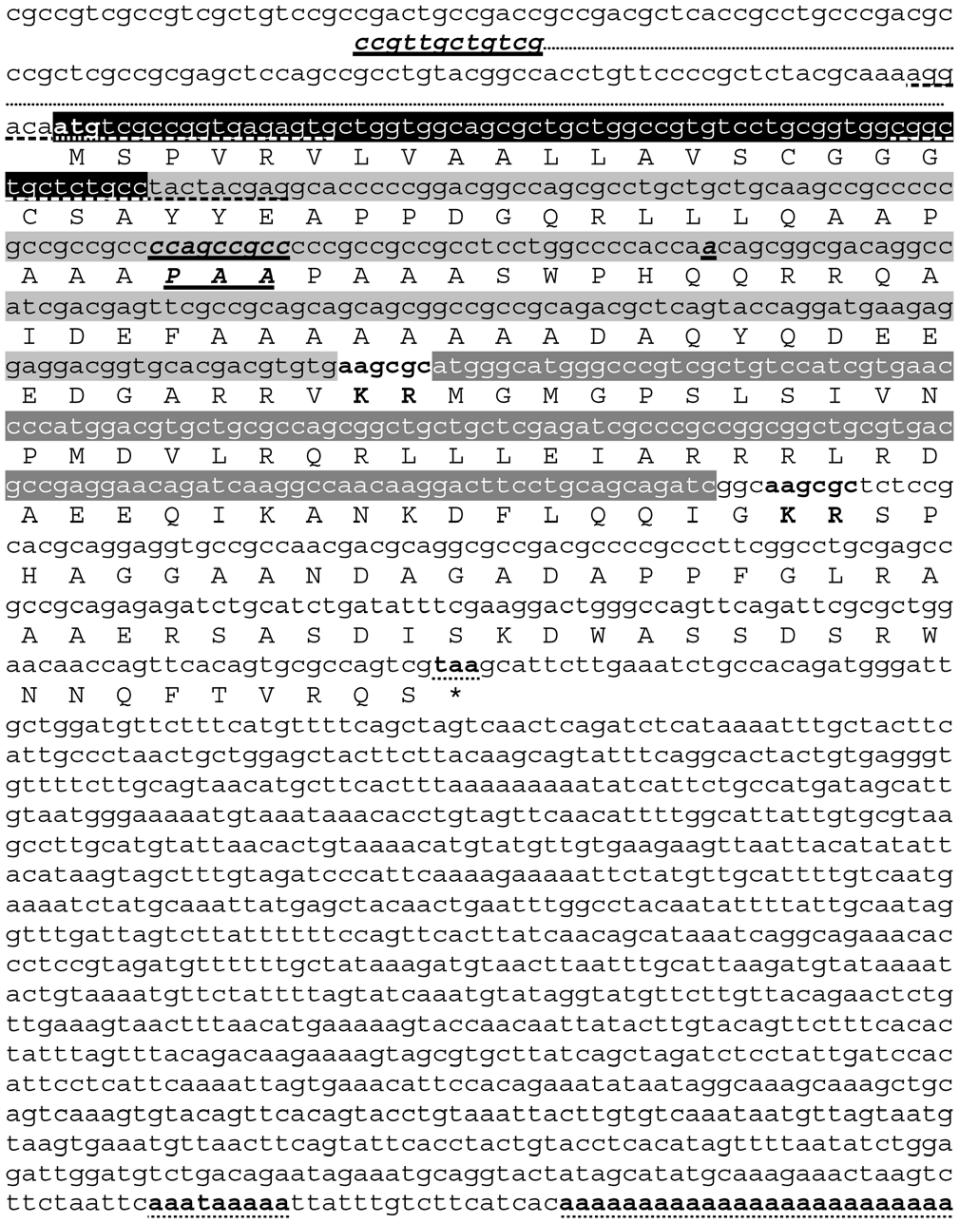
Sequences of the *S. gregaria* OMP-DH precursor cDNAs and the corresponding amino acid sequences. The longest of both precursor cDNAs (OMP-DH-L precursor cDNA, GenBank: JN591547) is shown here. Consecutively, the coding sequences for the signal peptide (white characters, highlighted in black), the OMP-peptide (black characters highlighted in light gray) and the CRF-like peptide (white characters, highlighted in dark gray) are represented. Predicted dibasic cleavage sites are denoted in bold. Start and stop codons, polyadenylation signal and poly(A) tail are denoted in bold and dotted underlined. The shorter precursor cDNA (*S. gregaria* OMP-DH-S precursor) differs from the longer one in the 5′UTR, a tripeptide insertion/deletion and a silent point mutation in the OMP-encoding sequence. All these differences are denoted in bold italics and single underlined. The 5′UTR sequence of the OMP-DH-S precursor is depicted under the 5′UTR of the other precursor. The primers used in the 3′RAcE-procedure are dashed underlined.

**Figure 2:**
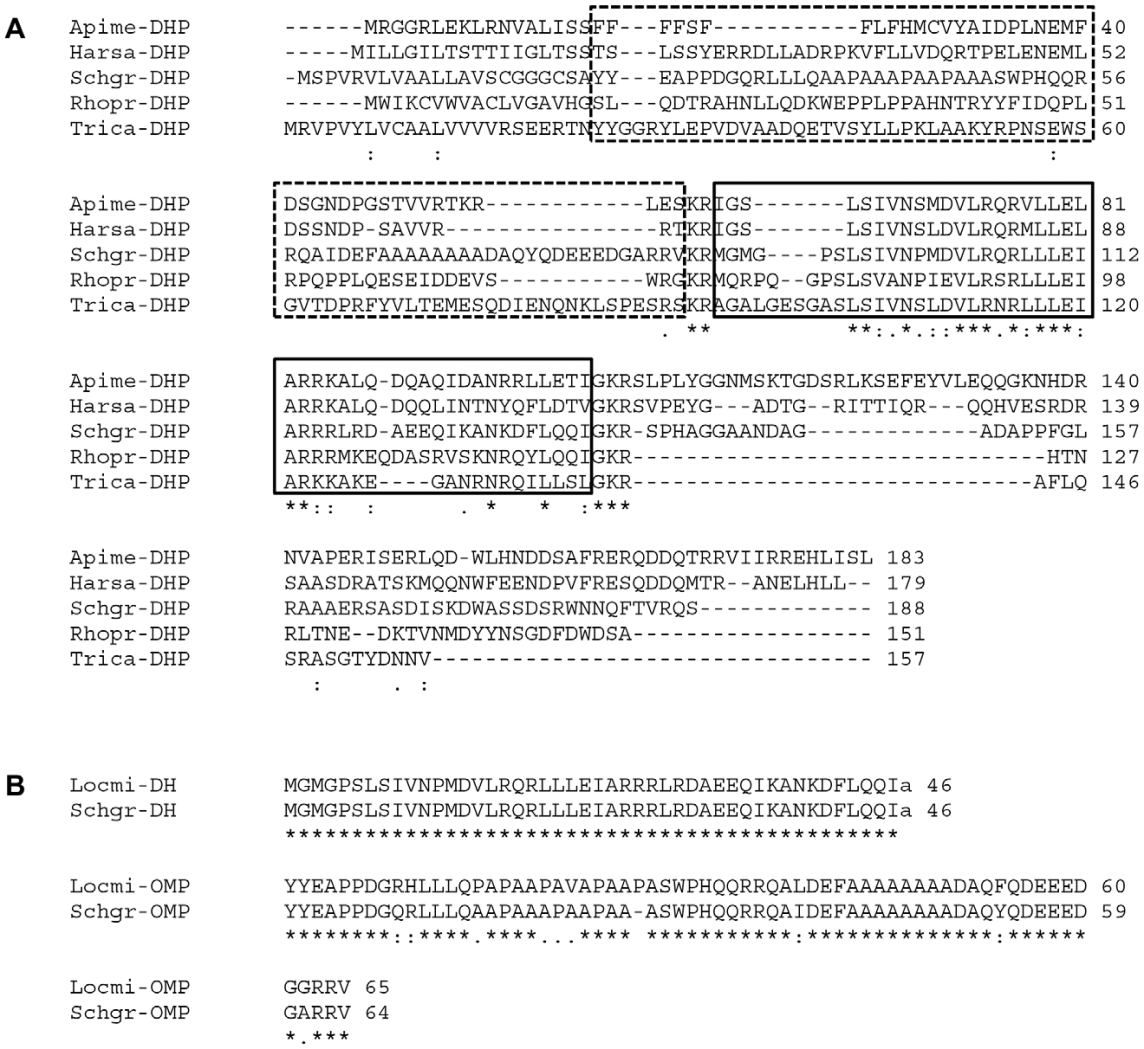
Analysis of sequence conservation. A) Multiple sequence alignment of the long *S. gregaria* OMP-DH precursor and several homologous insect precursors, performed with use of the ClustalW program (http://www.ebi.ac.uk/clustalw/), available from the European Bioinformatics Institute. Default parameters were used. The consensus is presented under each part of the alignment as follows: ‘*’ represents a position at which an amino acid is completely conserved, while ‘:’ and ‘.’ represent positions with lower degrees of conservation. The dashed boxes indicate the OMP-encoding region of the *S. gregaria* precursor (the region between the signal peptide and the first cleavage site of the *S. gregaria* precursor) and the corresponding region of the other precursors. The full boxes indicate the CRF-like DH coding region, flanked by dibasic cleavage sites (“(G)KR”). Abbreviations: *Apime*-DHP, *Apis mellifera* CRF-like DH precursor [16]; *Harsa*-DHP, *Harpegnathos saltator* CRF-like DH precursor (UniProtKB/TrEMBL: E2B7W2); *Schgr*-DHP, *S. gregaria* OMP-DH-L precursor (described in this paper); *Rhopr*-DHP, *Rhodnius prolixus* CRF-like DH precursor [5], UniProtKB/TrEMBL: F1AYR6; *Trica*-DHP, *Tribolium castaneum* CRF-like DH (47) precursor [17]. B) Alignment of the CRF-like peptides and OMPs from *Schistocerca gregaria* (*Schgr*-DH and *Schgr*-OMP) and *Locusta migratoria* (*Locmi*-CRF/DH and *Locmi*-OMP). Both CRF-like peptides are C-terminally amidated (“a”). Abbreviations: *Locmi*-CRF/DH, CRF-like DH from *L. migratoria* [6]–[7], UniProtKB/TrEMBL: P23465); *Schgr*-DH, CRF-like DH from *S. gregaria* (this paper); *Locmi*-OMP, Ovary Maturating Parsin from *L. migratoria* [19], UniProtKB/TrEMBL: P80045; *Schgr*-OMP: the longest Ovary Maturating Parsin isoform from *S. gregaria* [22].

In the *S. gregaria* EST database [24], we found two partial, highly similar, OMP-encoding precursor cDNA sequences. By means of 3′RAcE, we determined the 3′ part of these sequences. The complete cDNA sequences and the deduced amino acid sequences of the encoded precursors are represented in Figure 1. The precursors each code for one large *Schgr*-OMP isoform which is flanked at its C-terminus by a dibasic cleavage site. Remarkably, this cleavage site is followed by a CRF-like peptide, which is 100% identical to *Locmi*-CRF/DH, the CRF-like diuretic hormone of the migratory locust, *Locusta migratoria* [6]–[7] (Figure 2B). This sequence is again flanked at its C-terminus by a dibasic cleavage site (preceded by an amidation signal for the CRF-like peptide) followed by a sequence with no homology to other known sequences. When the amino acid sequences of both precursors are analyzed with the SignalP3.0 (Center for Biological Sequence Analysis, Technical University of Denmark, Lyngby, Denmark) [32], the most likely cleavage site for the signal peptide is located between position 22 and 23, the latter being the first amino acid of the previously purified long OMP-isoforms of *S. gregaria* [22]. Cleavage of the signal peptide combined with cleavage at the predicted dibasic cleavage site located directly at the C-terminus of the OMP sequences produces the long OMP isoforms. Both precursor cDNAs are very similar. Except for a partially different 5′UTR (5′untranslated region), they only differ from each other by a tripeptide insertion/deletion in the OMP sequences and a “silent” point mutation about 35 base pairs more downstream of this insertion/deletion. The resulting precursor proteins only differ from each other by the presence or absence of the tripeptide insertion in the OMP sequences.

### 2. Analysis of CRF/DH precursor sequence conservation

When the amino acid sequences of the *S. gregaria* OMP-DH precursors are compared to other known insect CRF-like DH precursors (Figure 2A, alignment with the *S. gregaria* OMP-DH-L precursor), the CRF/DH-coding region is found to be relatively well conserved together with its two flanking cleavage sites and the amidation signal. However, the precursor sequences situated between the signal peptide and the first cleavage site (encoding the OMPs in the *S. gregaria*-precursors) are not well conserved. All other currently known insect CRF/DH precursors (also those not included in Figure 2A) do not seem to code for OMP-like peptides. The OMPs (in the *S. gregaria* precursors) correspond to a pro-region of the other known CRF/DH-precursors.

When the CRF-like DH and OMP sequences from *L. migratoria* and *S. gregaria* are compared (Figure 2B; for *S. gregaria* the isoform most resembling to the *Locmi*-OMP was taken), we find a 100% identity for both CRF/DH sequences, while the OMP sequences show about 85% identity with most observed mutations being conservative. Also in locusts, CRF/DH is more conserved than OMP.

### 3. Transcript profiling

**Figure 3:**
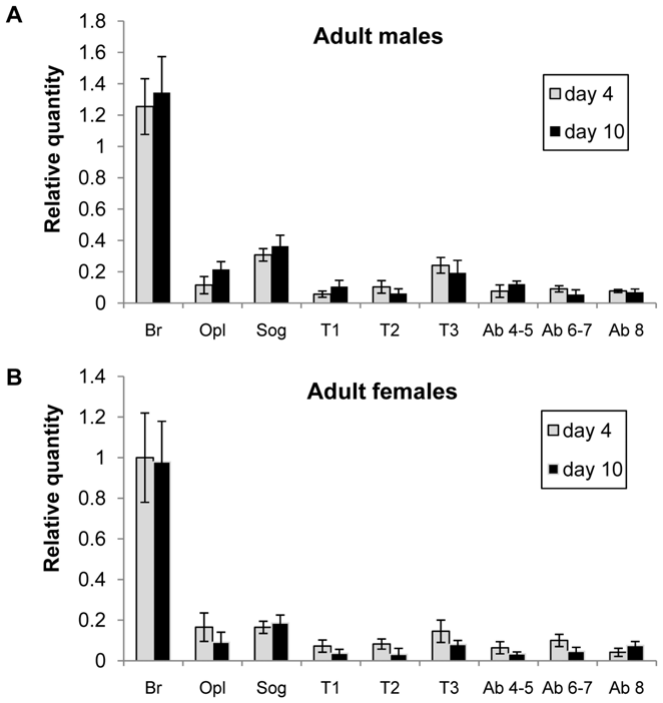
Graphical representation of relative OMP-DH transcript levels measured in the *S. gregaria* central nervous system. Transcript levels were determined on day 4 and day 10 of the adult stage in males (A) and females (B). Results were obtained by analyzing three independent pools of eight individuals per condition and are represented as means ± S.D. Normalization was performed according to Vandesompele *et al.* [26], using the geNorm applet. Abbreviations used on the X-axis: Br: brain, Opl: optic lobes, Sog: suboesophageal ganglion, T1: prothoracic ganglion, T2: mesothoracic ganglion, T3: metathoracic ganglion, Ab 4–5: abdominal ganglia 4 and 5, Ab 6–7: Abdominal ganglia 6 and 7, Ab 8: Abdominal ganglion 8.

By means of qRT-PCR, the tissue distribution of the *Schgr*-OMP-DH precursor transcripts was examined for both sexes, four and ten days after adult moult. The results are shown in Figure 3. In the four measured conditions (male day 4, male day 10, female day 4 and female day 10), a very similar distribution pattern was obtained. In all four conditions, the OMP-DH-precursor transcripts occur throughout the central nervous system (CNS), and their highest levels were measured in the brain. In the other measured tissues, OMP-DH mRNA was also detected, but transcript levels were much lower than the average transcript levels throughout the CNS, namely about 30 times lower for the fat body, midgut, Malpighian tubules and gonad samples and about 300 times lower for the other measured samples (the oesophagus, foregut, caeca, hindgut, flight muscle, epidermis, corpora cardiaca/corpora allata and the reproductive system without the gonads) (data not shown). For both sexes, no consistent differences were measured between the two different time points.

### 4. CRF/DH injection experiments

#### Effect of CRF/DH-injection on oocyte size

**Figure 4:**
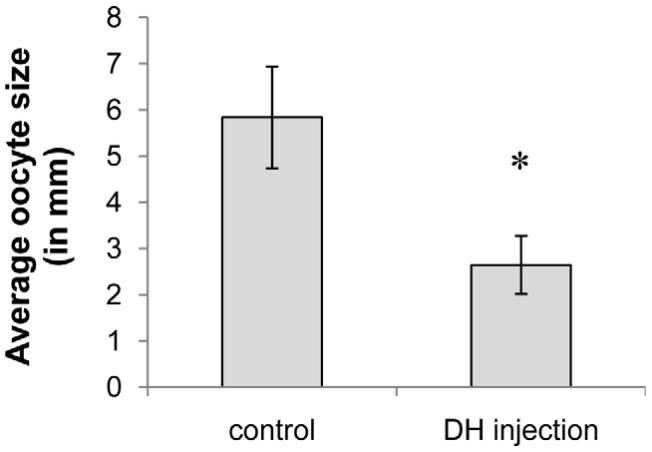
The effect of CRF/DH injection on oocyte growth in adult females. Animals were injected daily with 1 pmol CRF-like DH (dissolved in 4 µl Milli-Q® water) starting from day 1 until day 15 of the adult stage. Control animals were injected in the same way, but only with Milli-Q® water. The resulting average oocyte size (measured on day 15) is represented on the Y-axis. Results were obtained by analyzing more than 25 individuals per condition and are represented as means ± S.D. Asterisks indicate significant differences (*P*<0.01) between the experimental group and the control group (unpaired t-test).

Adult females were injected daily with 1 pmol of the CRF-like DH starting from day 1 until day 15 of the adult stage. Control animals were injected in the same way with Milli-Q® water. On day 15 of the adult stage, the oocyte size was measured. The average oocyte size was significantly smaller in the experimental group compared to the control group (Figure 4).

#### Effect of CRF/DH-injection on ecdysteroid levels

**Figure 5:**
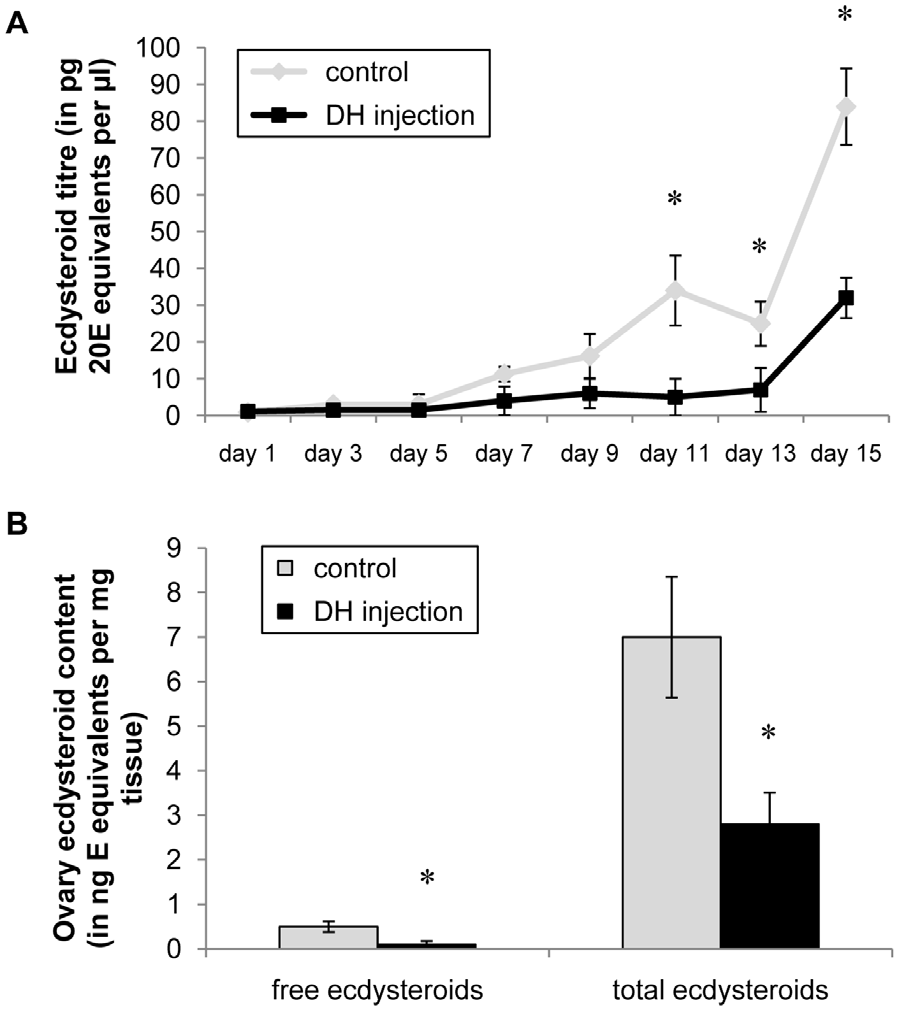
The effect of CRF/DH-injection on ecdysteroid levels in adult females. Ecdysteroid levels were determined for hemolymph samples (A) and ovary samples (B). Animals were injected daily with 1 pmol CRF-like DH (dissolved in 4 µl Milli-Q® water) from day 1 until day 15 of the adult stage. Control animals were injected in the same way, but only with Milli-Q® water. Hemolymph samples were taken on different time points and the ecdysteroid content was measured with the EIA. The resulting average ecdysteroid titers for the different time points are represented in figure 5A. On day 15, ovaries were dissected, ecdysteroids were extracted and measured with the enzyme immunoassay before (free ecdysteroids) and after enzyme treatment (“total” ecdysteroids). The resulting ecdysteroid content per mg tissue is represented in Figure 5B. Results were obtained by analyzing more than 25 individuals per condition and are represented as means ± S.D. Asterisks indicate significant differences (*P*<0.01) between the different experimental groups and control groups (unpaired t-test).

Adult females were injected daily with 1 pmol of the CRF-like DH from day 1 until day 15 of the adult stage. Control animals were injected in the same way with Milli-Q® water. At several time points during the experimental period, hemolymph samples were taken and ecdysteroid content was measured with EIA. On the last day of the experiment (day 15), ovaries were also dissected for determination of ecdysteroid levels. The effect of CRF/DH-injection on hemolymph ecdysteroid titers is shown in Figure 5A. During the first days of the experiment, no significant differences were measured in the hemolymph samples. However on day 11, 13 and 15, the animals injected with the CRF-like DH displayed significantly lower hemolymph ecdysteroid titers. They did not display the peak concentrations seen in the control animals on day 11 and 15, although the average concentration rose on day 15 (Figure 5A). The effect of CRF/DH-injection on the ovary ecdysteroid content is shown in Figure 5B. The ecdysteroid levels were measured before and after enzymatic treatment (which renders conjugated ecdysteroids detectable) and the average values are shown in Figure 5B. For both free ecdysteroids and total ecdysteroids (respectively before and after the enzymatic treatment), the measured ecdysteroid contents were significantly lower in the animals that were injected with the CRF-like DH compared to the control animals.

### 5. RNAi experiments

#### Effect of dsRNA-injection on oocyte size

**Figure 6:**
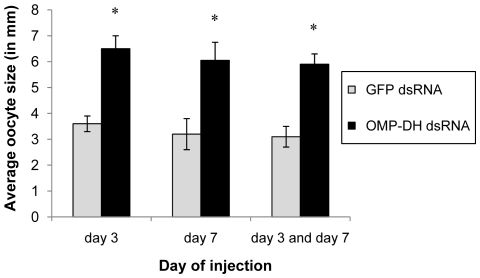
Effect of RNAi knockdown of the OMP-DH transcripts on oocyte size in adult females. Animals were injected with 4 µg OMP-DH dsRNA or 4 µg GFP dsRNA. The day of injection (day 3 and/or day 7) is represented on the X-axis. The resulting average oocyte size is represented on the Y-axis. Results were obtained by analyzing more than 20 individuals per condition and are represented as means ± S.D. Asterisks indicate significant differences (*P*<0.01) between the different experimental groups and control groups (One-way ANOVA).

To test the effect of the knockdown of OMP-DH transcript levels on oocyte size, adult females were injected with OMP-DH dsRNA or GFP dsRNA. Several injection schemes were applied but measurement of the oocyte size was always performed on day 12. All three injection experiments caused the same effect, namely a significantly larger oocyte size (Figure 6). The oocytes in the experimental groups approached their full-grown size and were nearly twice as big as in the control groups. The different injection schemes did not cause significantly different effects.

#### Effect of dsRNA-injection on ecdysteroid levels

**Figure 7:**
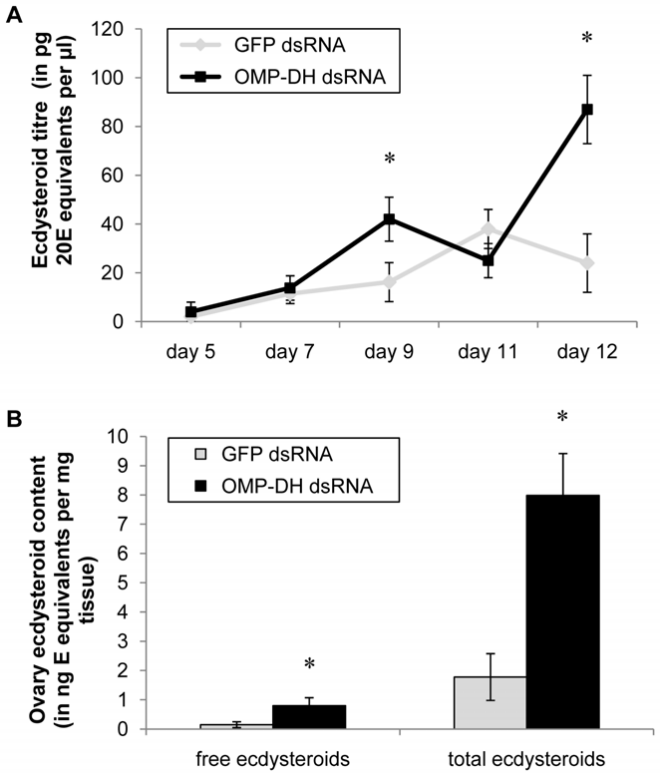
Effect of RNAi knockdown of the OMP-DH transcripts on ecdysteroid levels in adult females. Ecdysteroid levels were determined for hemolymph samples (A) and ovary samples (B). Animals were injected with 4 µg OMP-DH dsRNA or 4 µg GFP dsRNA on day 5. Hemolymph samples were taken on several time points until day 12. Samples were extracted and measured with the EIA. The resulting average ecdysteroid titers for the different time points are represented in Figure 7A. On day 12, ovaries were dissected, ecdysteroids were extracted and measured with the enzyme immunoassay before (free ecdysteroids) and after enzyme treatment (“total” ecdysteroids). The resulting ecdysteroid content per mg tissue is represented in Figure 7B. Results were obtained by analyzing about 25 individuals per condition and are represented as means ± S.D. Asterisks indicate significant differences (*P*<0.01) between the experimental and control groups (unpaired t-test).

Adult females received one single injection of OMP-DH dsRNA (or GFP dsRNA) at day five of the adult stage. At several time points after this dsRNA injection, hemolymph samples were taken. The extracted ecdysteroids were measured with the EIA. On the last day of the experiment (day 12), ovaries were also dissected for determination of ecdysteroid levels (both before and after enzymatic treatment). The effect of knockdown of the OMP-DH transcripts on hemolymph ecdysteroid titers is shown in Figure 7A. On day 5 (day of dsRNA injection), day 7 and day 11, ecdysteroid titers were almost equal for both conditions. On day 9 and day 12 however, significantly higher titers were measured in the experimental group compared to the control group. The effect of knockdown of the OMP-DH transcripts on ovary ecdysteroid content is shown in Figure 7B. For both free ecdysteroids and total ecdysteroids (respectively before and after the enzymatic treatment), the measured ecdysteroid contents were significantly higher in the ovaries of the experimental animals compared to the control ovaries.

### 6. Effect on food intake

**Figure 8:**
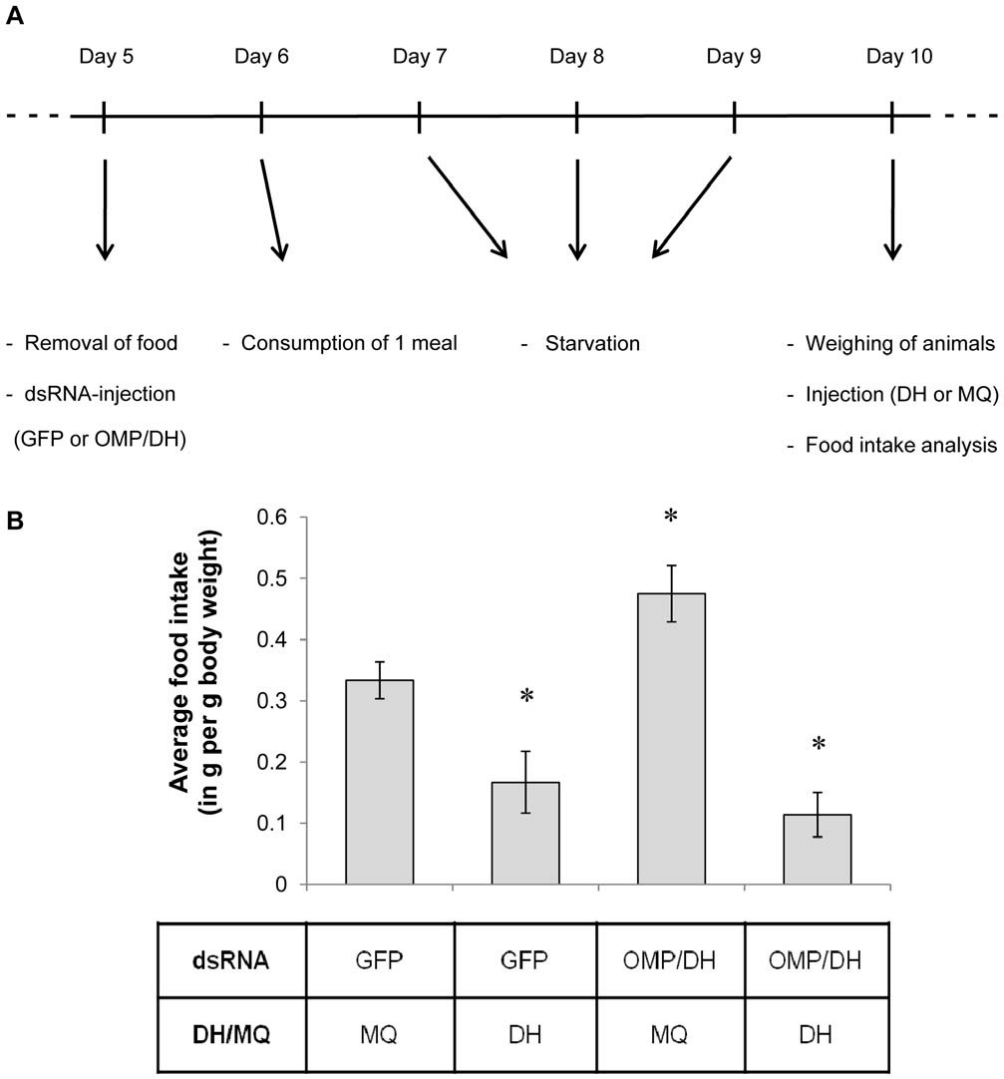
Effect of different experimental treatments on food intake. A) Time scheme adopted for the food intake experiment. B) The effects of CRF/DH-injection, RNAi knockdown of the OMP-DH precursors and “rescue” of this knockdown (by CRF/DH injection) were analyzed on food intake. Animals were injected with 4 µg OMP-DH dsRNA or 4 µg GFP dsRNA on day 5. On day 10 (after a few days of starvation), they were injected with CRF/DH (0.8 pmol in 4 µl Milli-Q® water) or Milli-Q® water (4 µl). The amount of food eaten by each animal was measured and corrected for evaporation and body weight. The average amount of food intake per g body weight is represented on the Y-axis. Results were obtained by analyzing about 30 individuals per condition and are represented as means ± S.D. Asterisks indicate significant differences (*P*<0.05) between the respective experimental group and the control group (One-Way ANOVA).

The effects of CRF/DH-injection, knockdown of the *Schgr*-OMP-DH precursors, as well as of a combination of both treatments, were analyzed on food intake. Animals injected with GFP dsRNA or OMP-DH dsRNA and CRF/DH or Milli-Q® water, were put in separate cages and were allowed to eat one meal. The workflow of this experiment is summarized in Figure 8A. The average amount of food eaten per gram body weight for the four different conditions (taking into account the weight loss of the leaves because of evaporation) is represented in Figure 8B. The OMP-DH dsRNA group (condition 3) shows a significantly higher food intake compared to the control group (condition 1). Both conditions that were injected with CRF/DH (with and without OMP-DH dsRNA, conditions 2 and 4 respectively), showed a significantly lower food intake compared to the control group. CRF/DH injection produced the opposite effect compared to the RNAi knockdown of the OMP-DH transcripts.

## Discussion

### 1. *S. gregaria* OMP-DH precursor transcript sequences

In the present paper, we describe the cloning of two highly similar OMP-DH-precursor cDNAs in *Schistocerca gregaria*. Both precursors encode one of both long OMP-isoforms, as well as a 46 amino acid member of the CRF-like DH peptide family (Figure 1). This peptide is identical to *Locmi*-CRF/DH, the CRF-like diuretic hormone of the migratory locust, *Locusta migratoria*, a species belonging to the same taxonomic family as *S. gregaria* (Figure 2B). Since homologues of this hormone display diuretic activity in diverse insect species [1], it is very likely that this peptide also regulates diuresis in *S. gregaria*. This paper is the first report on precursors both encoding OMP as well as CRF-like DH. By means of an immunohistochemistry study on the pars intercerebralis of *Locusta migratoria*, Tamarelle and coworkers already demonstrated that OMP immunoreactivity and CRF-like DH immunoreactivity are present in the same neuroendocrine cells [18]. The fact that both peptides originate from the same precursor, easily explains this observation.

Both cloned *S. gregaria* OMP-DH cDNAs are very similar and code for almost identical precursor proteins. Although the transcripts differ in their 5′UTR, a 9 bp insertion and one point mutation, the resulting prepropeptides only differ in the tripeptide (PAA) insertion/deletion within the OMP-coding region (Figure 1). This finding is in full agreement with the previously published sequences of the *Schgr*-OMP isoforms [22]. Interestingly, all observed differences between both characterized cDNA sequences occur in their 5′ part (*e.g.* a part of the 5′UTR is different), while the 3′part (with the 3′UTR) is completely identical for both precursors. Whether these transcripts originate from 2 recently diverged genes or represent splice variants from a single gene, remains to be investigated further.

As shown in Figure 2A, other currently known insect CRF/DH precursors do not seem to code for OMP-like molecules. OMP seems to correspond to a less conserved pro-region of the pre-pro-CRF/DH precursor (sometimes referred to as “cryptic peptide” [5]). This is consistent with the observations done by Richard and coworkers [23]. By means of immunohistochemistry, OMP-immunoreactivity was only found in the pars intercerebralis of Orthoptera from the Acrididae family, such as *L. migratoria* and *S. gregaria* [23]. Except for the *L. migratoria* and *S. gregaria* sequences, no OMP-like sequences can currently be found in the distinct genome, EST and protein/peptide databases. The exact nature of the compounds causing the immunoreactivity in the other acridid insects, has not been determined. On the other hand, the absence of immunoreactivity does not necessarily mean that no OMP-like peptides are present. Hence, the actual occurrence of OMP throughout different taxa can differ from the one suggested by Richard and coworkers [23]. Unfortunately, the lack of sequence information about the CRF/DH precursors in other acridid and non-acridid insect species, makes it difficult to conclude whether OMPs indeed only occur in the Acrididae.

It is not known whether the “cryptic peptides”, derived from the other insect CRF-like DH precursors, have a function in these insects. Since this precursor region is not conserved, it seems unlikely that this region would play an “OMP-like” role in the physiology of non-locust species. In the red flour beetle *Tribolium castaneum*, a part of this precursor region (but also the C-terminal precursor part) was found *in vivo* [17], but no physiological function could be attributed. Also in vertebrate CRF-precursors, a pro-region is situated between the signal peptide and the CRF-encoding precursor region [33]–[34]. It is however not clear if this region has any physiological or endocrinological function.

### 2. *Schgr*-OMP-DH transcript profiling

The results of the transcript profiling experiment are in accordance with previously published immunological data obtained in *L. migratoria*. Both for the CRF/DH and OMP, immunoreactivity was found throughout the CNS of adult *L. migratoria*, with the highest immunoreactivity observed in the brain [11], [35], [36]. In other (non-orthopteran) insect species, CRF-like DH immunoreactivity was also detected in the brain and other parts of the CNS [37]–[39]. As described in this study, *Schgr*-OMP-DH precursor transcripts were also found in tissues not belonging to the CNS, although to a much lesser extent. *Schgr*-OMP-DH transcripts were detected in the Malpighian tubules, midgut and hindgut. This appears logical since the Malpighian tubules and the hindgut are involved in excretion and diuresis, while ion and water transport also occurs across the midgut. Interestingly, in *L. migratoria*, CRF-like DH immunoreactivity was found in granules of endocrine cells in the ampullae at the border of the Malpighian tubules and the gut, indicating that this hormone is probably produced in these cells [40] and in *S. gregaria*, a similar situation may occur. The transcript levels (as measured in the hindgut, midgut and Malpighian tubules, but also in other non-CNS tissues) suggest that there may also be other cells expressing CRF/DH in *S. gregaria*, however detection of transcript may not necessarily mean that the resulting peptides are also present and functional. In larvae of the tobacco hornworm *Manduca sexta*, CRF/DH encoding precursor transcripts were also detected in the gut and Malpighian tubules [13].

### 3. RNAi procedure

To further investigate the role of the *Schgr*-OMPs and the *Schgr*-CRF-related diuretic hormone in *S. gregaria*, RNAi studies were performed. In order to verify the expected “knockdown” effect of the injection of the dsRNA, the OMP-DH transcript levels were measured by qRT-PCR five and seven days after injection (Figure S2). In both cases, a very robust knockdown (>90%) was observed in the brain, optical lobes and suboesophageal ganglia. The knockdown efficiency was very similar at both analyzed time points. This means that a stable knockdown of the OMP-DH transcript levels can be obtained for a considerable period by means of a single injection of OMP-DH dsRNA in the hemocoel. Similar systemic RNAi efficiencies were already successfully obtained in our lab with other locust transcripts ([41]–[43] and several unpublished results). This indicates that this dsRNA-induced post-transcriptional silencing mechanism can be employed for generating an efficient knock down of a given transcript in locusts.

### 4. CRF-like DH reduces food intake

We demonstrated that injection of CRF-like DH prior to the meal caused a significant reduction in food intake. On the other hand, feeding was stimulated when animals were injected with OMP-DH dsRNA, while injection with CRF/DH overruled this effect and again caused a clear reduction in food intake (Figure 8). The observed effects on food intake are in line with previous reports on the antifeedant biological activities of *Locmi*-CRF/DH in *L. migratoria*. Injection of this CRF/DH, as well as analogues and truncated forms, into nymphs of *L. migratoria* increased the latency to feeding and reduced meal duration [8]–[9], while CRF/DH was shown to be released progressively during the meal [10], [11]. Therefore, it was suggested that CRF/DH may regulate satiety and signal the end of feeding in locusts [8], [12], probably together with some other factors, such as sulfakinins [44], [45]. Moreover, injection of the *M. sexta* CRF-like DH in larvae of the moth *Heliothis virescens* also caused decreased food consumption (and increased weight loss), although high doses of the peptide were needed [46]. When neonates of *Manduca sexta* were fed leaf discs treated with the shorter *M. sexta* CRF-like diuretic hormone (the *Manduca* diuresin, a 30 amino acid peptide), they exhibited reduced food consumption [47].

In locusts, the effect of CRF/DH on food intake was suggested to possibly result (in part) from a decrease in peripheral sensitivity to food stimuli, caused by the closure of the pores of taste sensilla on the mouthparts [12]. Gustatory stimuli, but also olfactory stimuli, are important in regulating and adjusting insect feeding behavior, since insects use these stimuli to localize food and to assess food quality [48]–[51]. Some reports suggest that modulation of chemosensory perception can influence foraging and feeding behavior [52], [53]. In *Drosophila melanogaster*, chemosensory perception is modulated *in vivo* by neuropeptides, while some of these peptides also affect feeding behavior [54]–[58]. Short Neuropeptide F mediates odor-driven food search [56]. Leucokinin modulates chemosensory responses, but also affects meal size [57]–[58]. These reports indicate that several neuropeptides indeed can influence chemosensory perception, which may affect appetite and feeding behavior in insects, as suggested for CRF-like DH in locusts [12]. Interestingly, a 15-residue C-terminal fragment of *Locmi*-CRF/DH (consisting of residues 32–46) also displays clear antifeedant activity in locusts, although this peptide only shows very weak *in vitro* diuretic activity on Malpighian tubules (at very high concentrations) [9]. This is consistent with the observation that residues at the N-terminus of the *Locmi*-CRF/DH are important for activating the receptor on the Malpighian tubules [59]. It seems that the structure-activity requirements of *Locmi*-CRF/DH for mediating diuresis, differ from those necessary for mediating feeding behavior, suggesting that both activities are regulated by functionally different signaling systems [9]. In this respect, it is important to note that in *D. melanogaster*, two CRF/DH receptors were found with clearly distinct signaling properties and peptide sensitivities [60], [61]. While both receptors are clearly expressed in the brain, only one of both displays a distinct expression in the Malpighian tubules, which led to the suggestion that there might be other functions of CRF/DH in the fruit fly, in addition to osmoregulation [61]. Analysis of genomic data suggests that (some) other insect species also have more than one CRF/DH receptor. Nevertheless, further functional investigations are necessary to confirm this idea and to further analyze CRF/DH-induced signaling processes in insects.

Remarkably, CRF also displays antifeedant activity in vertebrates [62]–[64], where it is a component of the hypothalamic-pituitary-adrenal axis and mainly mediates stress responses [65]. Although both CRF and CRF/DH have a negative effect on feeding, their general physiological roles seem rather different.

### 5. CRF-like DH inhibits oocyte growth and reduces ecdysteroid levels

As seen in Figure 4, female adults injected with the CRF-like DH displayed a smaller oocyte size, as well as lower ecdysteroid levels in the hemolymph and the ovaries (Figure 5). The knockdown of the OMP-DH precursors resulted in the opposite outcome (Figure 6 and 7), suggesting that the observed effects were caused by influencing the CRF-like DH signaling system. The exact mode of action of CRF/DH (direct or indirect) in causing these *in vivo* effects is not fully clear yet. It is possible that the antifeedant activity of the CRF/DH injection has indirectly caused attenuating effects on oocyte growth and/or ecdysteroid levels. Conversely, RNAi knockdown of the OMP-DH precursors will probably have stimulated the locusts to eat more, which may have resulted in the acquisition of more energy and nutrients in support of anabolic processes, such as vitellogenesis. Several reports have been made about effects of diet and food quality on locust reproduction [66]–[68], while the same effect is presumed to occur in nature when locusts start to reproduce massively when food sources become more abundant and nutritious after periods of rainfall [25]. Also in other insect species, similar observations have been made (*e.g.* for cockroaches [69]; for butterflies [70]; and for bugs: [71]). A higher nutrient uptake may not only stimulate vitellogenesis and oocyte growth, but might also affect other anabolic processes (related to reproduction), such as ecdysteroidogenesis. Tawfik and coworkers measured the ovary ecdysteroid content and hemolymph ecdysteroid titers in function of the first female reproductive cycle in *S. gregaria* [28], [31]. When we compare their data with our observations (Figure 5 and 7), it appears that the ecdysteroid levels and contents of the experimental animals can be interpreted as precocious (RNAi) or delayed (CRF/DH injection) with regard to the normal situation (or control condition). Although its antifeedant activity may have indirectly affected oocyte growth and ecdysteroidogenesis, it is possible that the CRF-like DH exerts direct biological activities that have contributed to these *in vivo* effects. Perhaps, these activities may even be more important in causing the effects. The observed *in vivo* effects of CRF/DH may therefore be part of a wider repertoire of neurohormonal fine-tuning activities, constituting an integrating control system that not only affects food intake and excretion, but also anabolic processes like vitellogenesis and ecdysteroidogenesis, following a meal. Further investigation will be needed to clarify the exact regulatory hierarchy causing the observed effects.

In vertebrates, CRF also seems to display effects on vertebrate reproductive physiology, *e.g.* by negatively affecting the secretion of Gonadotropin Releasing Hormone (GnRH), Luteinizing hormone (LH) and Follicle Stimulating hormone (FSH) in some vertebrates [72], [73]. Again, it seems difficult to say whether this activity is homologous or analogous to the activities of CRF-like DH described in this study.

The insect ovaries are the main production site for ecdysteroids in female adults. A large part of these ecdysteroids is stored as conjugates in the ovaries and will be used during embryonic development [74]–[76]. Others are leaked into the hemolymph. The amount of conjugated ecdysteroids in the ovaries is higher than the amount of free ecdysteroids (as can be concluded from the measurements before and after enzyme treatment to convert the conjugated ovary ecdysteroids, Figure 5B and 7B), as was observed previously in *S. gregaria* [28], [77]. The exact role of the hemolymph ecdysteroids in adult female locusts remains uncertain. Studies on *L. migratoria* suggested that 20E influences vitellogenin synthesis [21], while a study on the lubber grasshopper *Romalea microptera* concluded that hemolymph ecdysteroids did not affect vitellogenesis in that species [78]. In any case, ecdysteroids are synthesized by the ovaries (follicle cells) and incorporated in growing oocytes during vitellogenesis, explaining temporal correlations of their synthesis (and appearance) with oocyte growth during the gonotrophic cycle of the locust.

When solely keeping in mind the *in vivo* effects of OMP injection (stimulation of oocyte growth and precocious ecdysteroid hemolymph peaks [19], [21], [22], one would intuitively expect that the OMP-DH dsRNA injection would cause an inhibition of oocyte growth as well as lower ecdysteroid titers (or delayed ecdysteroid peaks). However, exactly the opposite was observed (Figure 6 and 7). At present, it is not clear how OMP may have affected this result. Our observations can be explained by the action of the CRF-like DH. As previously mentioned, the nicely opposing effects of dsRNA and CRF-like DH injections (described in this study) suggest that the RNAi knockdown indeed caused lower CRF-like DH levels (and probably also lower OMP levels). Likely, the effect of reduced CRF/DH signaling on vitellogenesis and ecdysteroid levels simply overruled the effect of reduced OMP signaling, resulting in the observed effects. Another possibility is that *in vivo* injection of OMP molecules [19]–[22] may induce a negative feedback which leads to a lower synthesis and/or release of both OMP and CRF/DH. As a result of this, OMP injection would then lead to a reduced level of CRF/DH causing a similar situation as in RNAi studies. The OMPs would then act as a “monitor peptide” for CRF/DH (Figure S3). Since both neurohormones originate from a single precursor, their synthesis is directly linked and situated in the same cells. Therefore, one possible hypothesis is that OMP may act as a monitor peptide, which exerts a negative feedback control on the synthesis and/or release of both OMP and CRF/DH and/or on the biological activity of CRF/DH. Further investigation is needed to clarify the functional relationship between CRF/DH and OMP.

## Supporting Information

Figure S1 **Production of the “sense” and “anti-sense” templates used in dsRNA production.** A) By means of PCR, the coding sequence was amplified and a unique restriction site was added to each side of this DNA fragment. B) This PCR product was cloned in “sense” and “anti-sense” orientation in a pCR™4-TOPO® vector, downstream of the T7 promoter. To enhance the transcription efficiency, the resulting templates were linearized. This was done by specific cleavage at the restriction site situated immediately downstream of the coding sequence. After transcription, the “sense” and “anti-sense” RNA strands were annealed, purified and used for injection.
(TIF)

Figure S2 **Effect of OMP-DH dsRNA injection on the** ***Schgr*** **-OMP-DH transcript levels in adult females.** Adult females were injected with GFP dsRNA or OMP-DH dsRNA. Transcript levels were determined in different tissues five days (A) and seven days (B) after dsRNA injection. Results were obtained by analyzing three independent groups of ten individuals per condition and are represented as means ± S.D. Abbreviations used on the X-axis: Br: brain, OpL: optic lobes, SoG: suboesophageal ganglion. Asterisks indicate a significant difference (*P*<0.05) in the respective tissue between the two treatments (linear regression analysis).
(TIF)

Figure S3 **Schematic representation of hypotheses that may explain the opposite effects of OMP and CRF/DH.** Since OMP and CRF/DH originate from a single precursor, their synthesis is directly linked and situated in the same cells. OMP may act as a monitor peptide, which exerts a negative feedback control on the synthesis and/or release of both OMP and CRF/DH and/or on the biological activity of CRF/DH (a). Alternatively, OMP may act separately and generate effects that can be overruled by CRF/DH (b).
(TIF)
