# Supplementary figures and images for: CRF-Like Diuretic Hormone Negatively Affects Both Feeding and Reproduction in the Desert Locust, Schistocerca gregaria
Source: PLoS One. 2012 Feb 20;7(2):e31425. doi: 10.1371/journal.pone.0031425 (PMC3282710; doi:10.1371/journal.pone.0031425)

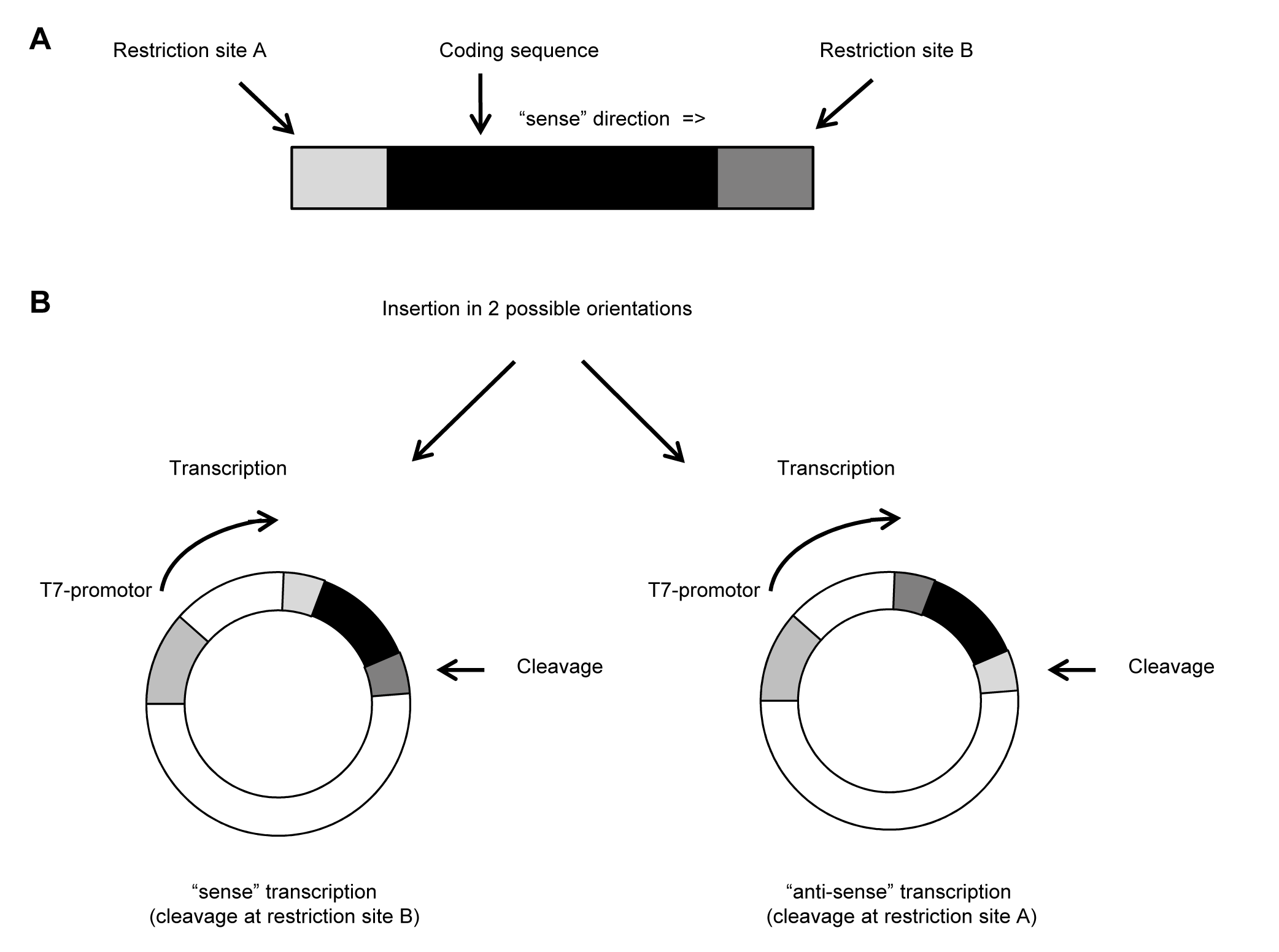

Supplement: Figure S1 — Production of the “sense” and “anti-sense” templates used in dsRNA production. A) By means of PCR, the coding sequence was amplified and a unique restriction site was added to each side of this DNA fragment. B) This PCR product was cloned in “sense” and “anti-sense” orientation in a pCR™4-TOPO® vector, downstream of the T7 promoter. To enhance the transcription efficiency, the resulting templates were linearized. This was done by specific cleavage at the restriction site situated immediately downstream of the coding sequence. After transcription, the “sense” and “anti-sense” RNA strands were annealed, purified and used for injection. (TIF) [file pone.0031425.s001.tif]

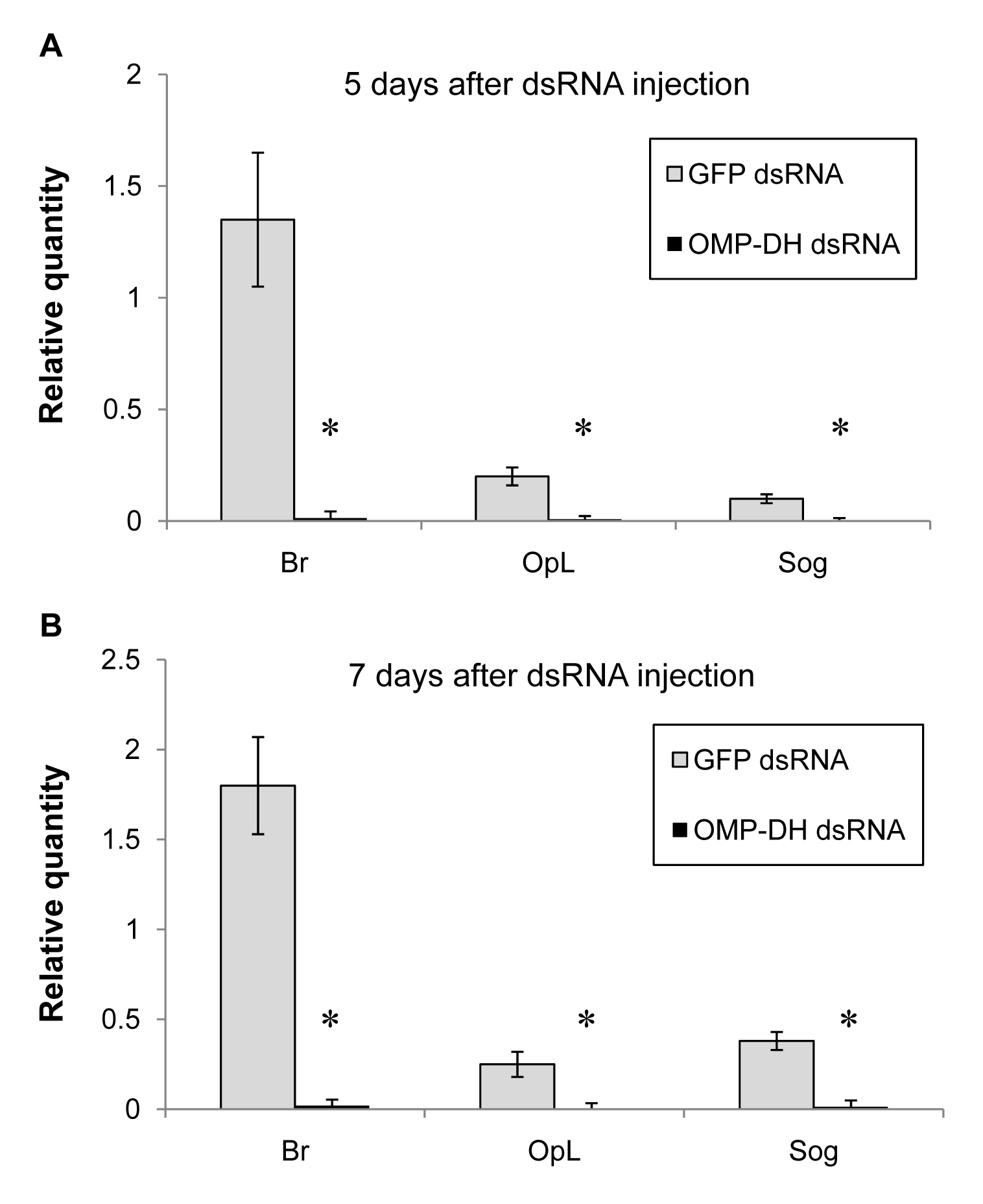

Supplement: Figure S2 — Effect of OMP-DH dsRNA injection on the Schgr -OMP-DH transcript levels in adult females. Adult females were injected with GFP dsRNA or OMP-DH dsRNA. Transcript levels were determined in different tissues five days (A) and seven days (B) after dsRNA injection. Results were obtained by analyzing three independent groups of ten individuals per condition and are represented as means ± S.D. Abbreviations used on the X-axis: Br: brain, OpL: optic lobes, SoG: suboesophageal ganglion. Asterisks indicate a significant difference (P<0.05) in the respective tissue between the two treatments (linear regression analysis). (TIF) [file pone.0031425.s002.tif]

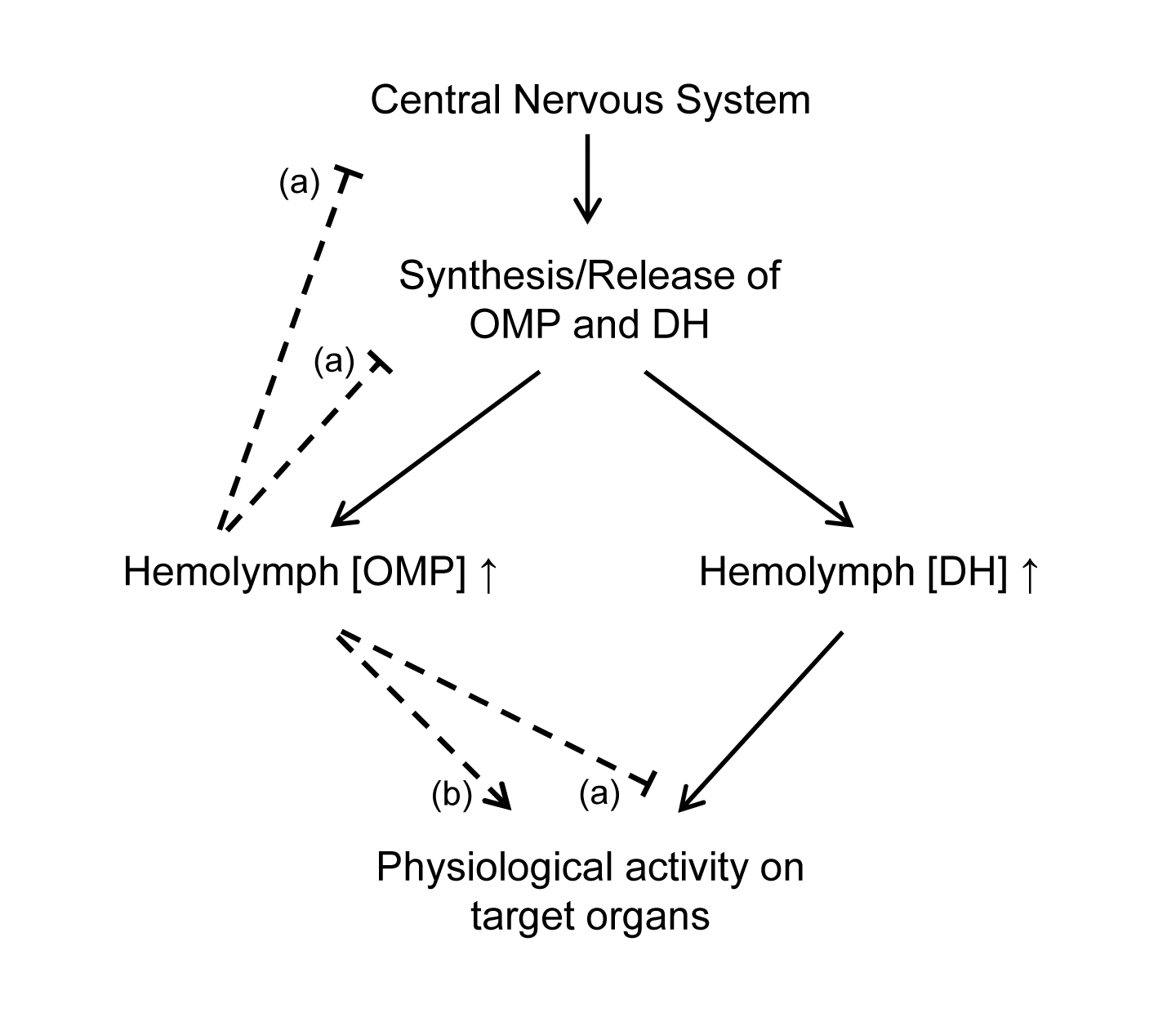

Supplement: Figure S3 — Schematic representation of hypotheses that may explain the opposite effects of OMP and CRF/DH. Since OMP and CRF/DH originate from a single precursor, their synthesis is directly linked and situated in the same cells. OMP may act as a monitor peptide, which exerts a negative feedback control on the synthesis and/or release of both OMP and CRF/DH and/or on the biological activity of CRF/DH (a). Alternatively, OMP may act separately and generate effects that can be overruled by CRF/DH (b). (TIF) [file pone.0031425.s003.tif]
